# The Third Report of the Royal Commission on Vivisection

**Published:** 1907-11-02

**Authors:** 


### SPECIAL ARTICLE.

### THE THIRD REPORT OF THE ROYAL COMMISSION ON VIVISECTION.

The Royal Commissioners on Vivisection have published a Third Report, together with an Appendix containing the evidence given before them during April, May, June and July of the present year. Among the witnesses examined were Lord Justice Fletcher Moulton, the Hon. Stephen Coleridge (representing the National Anti-Vivisection League), the Rev. L. S. Lewis (representing the Church Anti-Vivisection League), Sir Lauder Brunton, Mr. Henry Morris (President of the Royal College of Surgeons), Dr. Martin (Director of the Lister Institute), and Miss Lind af-Hageby.

The evidence of the medical witnesses was chiefly directed to showing that medical science has made very great progress as the result of experiments on living animals, and that if these experiments be prohibited further advance will be seriously hampered or even rendered impossible. We imagine that few of our readers, who are acquainted with the facts, will dispute the truth of this contention. We do not, therefore, propose to discuss the point further, but will pass on to a consideration of the evidence of the anti-vivisectionist witnesses.

From this it appears that the anti-vivisectionists base their objections to experiments on animals on one or other of two main arguments. Some, who may be regarded as extremists, maintain that man has no right to make use of the lower animals for scientific purposes. They refuse to admit that these practices can be justified in the least degree by any useful discoveries that may spring from them, and they often go so far as to deny that any such discoveries have been made. It is clear that those who take up this position will be satisfied with nothing less than the prohibition of all experiments on living animals. Others, however, adopt a less uncompromising attitude. They object to these experiments only in so far as they cause pain to the animal, and would consent to their performance if pain could be avoided.

Mr. Stephen Coleridge, who represents the largest of the Anti-Vivisectionist Societies, and is commonly regarded as the protagonist of the movement, seems to fall within the latter class. In his opening statement, Mr. Coleridge says: "My Society desires to see vivisection totally abolished by law, but will accept any measures that have for their object the amelioration of the condition of vivisected animals"; and again, "We think that to bring in a Bill for the total abolition of all vivisection in the present condition of public opinion would be a waste of time." When asked for his personal opinion, he replies, "My objection to vivisection begins and is centred in the question of pain. . . . If the pain be really and truly eliminated from vivisection, I have no objection." Mr. Coleridge brings to the notice of the Commission a Bill, approved by his Society, containing certain provisions which, he believes, would remove most of the features of the present system of control to which he takes exception. But, before reviewing the heads of this Bill, it may be advisable to describe briefly the existing regulations, with which some of our readers are, perhaps, unfamiliar.

In accordance with the Act 39 and 40, Vict., a person shall not perform on a living vertebrate animal any experiment calculated to give pain, unless he has received a license from the Home Secretary. This license allows the holder to perform experiments with a view to the advancement of physiological knowledge or of knowledge useful for the saving of life or the alleviation of suffering, provided that the animal is kept under an anæsthetic during the whole of the operation, and that, if pain is likely to continue after the operation, or if serious injury has been inflicted, the animal must be killed before it regains consciousness. The powers conferred by the license may be extended by the granting of certificates. Certificate A permits the licensee to dispense with anæsthetics under certain conditions; and this certificate covers the inoculation experiments which form such a large percentage of all experiments done under the Act. Certificate B cancels the obligation to kill the animal before it recovers from the anæsthetic, and applies chiefly to physiological experiments. Certificate C allows the performance of experiments to illustrate lectures. The remaining certificates, four in number, permit experiments on horses, asses, dogs and cats, the use of which is illegal under the license alone. Applications for a license must be endorsed by a President of one of the Royal Societies or Colleges, and by a Professor of Medicine or some kindred subject in one of the recognised teaching institutions of the United Kingdom. The license must be renewed annually, and without a license the certificates are of no avail.

Mr. Coleridge describes his Bill as an amendment to, rather than a repeal of, the present Act; and his proposed alterations fall under three main heads. In the first place, he would limit the range of experiments. Mr. Coleridge, in common with other anti-vivisectionists, falls especially foul of the physiological experiments performed under Certificate B. He believes that the animals suffer severe pain as the result of these experiments, and consequently he would entirely do away with certificate B. He would do away with certificate C, thus prohibiting the performance of experiments for teaching purposes. He would also modify the conditions of certificate A, and provide that an animal inoculated under this certificate should be either killed or kept under an anæsthetic, as soon as it begins to suffer serious pain as the result of inoculation. In the second place he would enlarge the scope of action of the inspectors under the Act, by requiring that one of them should be present at every serious physiological operation. Finally, in the appointment of licensees and inspectors, he would require special regard to be paid to the applicant's reputation for humanity.

We quote Mr. Coleridge's proposals, not in order to criticise them, for we are quite content to abide by the future recommendations of the Commission, but to show wherein those whom Mr. Coleridge represents regard the present Act as deficient. By way of comment we will merely say that, although inclined to agree to the restriction of experiments for teaching purposes under certificate C, we are convinced that the abolition of certificate B would severely check the advancement of science. The suggestion as to proving the humanity of licensees and inspectors seems an unattainable council of perfection, and Mr. Coleridge's idea that this might be achieved by requiring every applicant for a license to produce a certificate of humanity signed by a Justice of the Peace or a minister of religion strikes us as entirely ridiculous.

Miss Hageby, whose evidence appears in this report, is an extreme type of anti-vivisectionist. She considers that all experiments on animals are morally wrong, and that nothing which she would call useful has been discovered thereby. She objects to vaccination, and to the treatment of diphtheria by antitoxin. Miss Hageby will be remembered as the author of "The Shambles of Science," a book which attained a considerable notoriety in the trial of the action Bayliss *v.* Coleridge; and the issue of that trial does not seem to have deterred her from giving utterance to the most unwarrantable mis-statements in support of her crusade. When the anti-vivisectionists take their stand on the argument that experiments on animals are ethically wrong, it is a matter of opinion, and they have a right to an opinion as much as anyone else; but when they deny that these experiments have been of any benefit to mankind it is no longer a matter of opinion, but of fact, and the facts which they dispute are held to be proved beyond doubt by all qualified to judge.

Unfortunately the whole question of vivisection is one that appeals primarily to the emotions, and is apt to be judged by an emotional standard rather than submitted to the true test of rational consideration. The arguments employed by the leaders of the anti-vivisection movement are extremely plausible and likely to pass unquestioned amongst those who have no knowledge of the facts; they undoubtedly serve to win supporters to the cause, as well as to stir up a great deal of resentment against the supposed barbarity of the scientists. But we do not for a moment believe that the members of the various anti-vivisection societies, if both sides of the question could be put before them in a dispassionate and impartial way, would continue to endorse the vehement denunciation of the whole system of animal experiment that we are wont to hear from the majority of their representatives; and we are convinced that most of the ill-feeling and misunderstanding which surrounds this controversial subject is due to ignorance and prejudice.

